# Synthesis and crystal structure of 3-(adamantan-1-yl)-4-(2-bromo-4-fluoro­phen­yl)-1*H*-1,2,4-triazole-5(4*H*)-thione

**DOI:** 10.1107/S2056989020000092

**Published:** 2020-01-10

**Authors:** Alaa S. Abdelrazeq, Hazem A. Ghabbour, Ali A. El-Emam, Doaa Ahmed Osman, Santiago Garcia-Granda

**Affiliations:** a Department of Medicinal Chemistry, Faculty of Pharmacy, University of Mansoura, Mansoura 35516, Egypt; b Department of Physical and Analytical Chemistry, Faculty of Chemistry, Oviedo University-CINN, Oviedo 33006, Spain

**Keywords:** crystal structure, adamantane, triazole

## Abstract

In the title compound, C_18_H_19_BrFN_3_S, the 1,2,4-triazole ring is nearly planar with a maximum deviation of −0.009 (3) and 0.009 (4) Å, respectively, for the S-bound C atom and the N atom bonded to the bromo­fluoro­phenyl ring. The phenyl and triazole rings are almost perpendicular to each other, forming a dihedral angle of 89.5 (2)°. In the crystal, the mol­ecules are linked by weak C—H⋯π(phen­yl) inter­actions, forming supra­molecular chains extending along the *c-*axis direction. The crystal packing is further consolidated by inter­molecular N—H⋯S hydrogen bonds and by weak C—H⋯S inter­actions, yielding double chains propagating along the *a*-axis direction. The crystal studied was refined as a racemic twin.

## Abstract 2

The crystal structure of this novel adamantan-based compound is built up by organic chains formed and stabilized *via* C—H⋯π, N—H⋯S and C—H⋯S inter­actions.

## Chemical context   

Adamantane derivatives are currently receiving considerable inter­est for their diverse biological activities (Liu *et al.*, 2011 ▸; Lamoureux & Artavia, 2010 ▸). Numerous adamantane-based drugs have been developed as anti­viral (Davies *et al.*, 1964 ▸; Togo *et al.*, 1968 ▸; Rosenthal *et al.*, 1982 ▸; El-Emam *et al.*, 2004 ▸; Burstein *et al.*, 1999 ▸; Balzarini *et al.*, 2009 ▸), anti­cancer (Sun *et al.*, 2002 ▸; Min *et al.*, 2017 ▸), anti­diabetic (Villhauer *et al.*, 2003 ▸ & Augeri *et al.*, 2005 ▸), anti-Parkinsonian (Schwab *et al.*, 1969 ▸), anti-Alzheimer’s (Bormann, 1989 ▸) and anti­psychotic (Abou-Gharbia *et al.*, 1999 ▸) agents. In addition, several adamantane-based analogues have been shown to possess promising bactericidal (Protopopova *et al.*, 2005 ▸; El-Emam *et al.*, 2013 ▸; Kadi *et al.*, 2010 ▸; Al-Abdullah *et al.*; 2014 ▸; Al-Deeb *et al.*, 2006 ▸) and fungicidal (Omar *et al.*, 2010 ▸) activities. On the other hand, 1,2,4-triazole derivatives have been reported to possess significant anti-inflammatory (Navidpour *et al.*, 2006 ▸) and anti­bacterial activities (Almajan *et al.*, 2009 ▸). Based on the diverse biological activities of adamantane and 1,2,4-triazole derivatives, we synthesized the title 1,2,4-triazole-adamantane hybrid derivative **I** as potential chemotherapeutic agent.
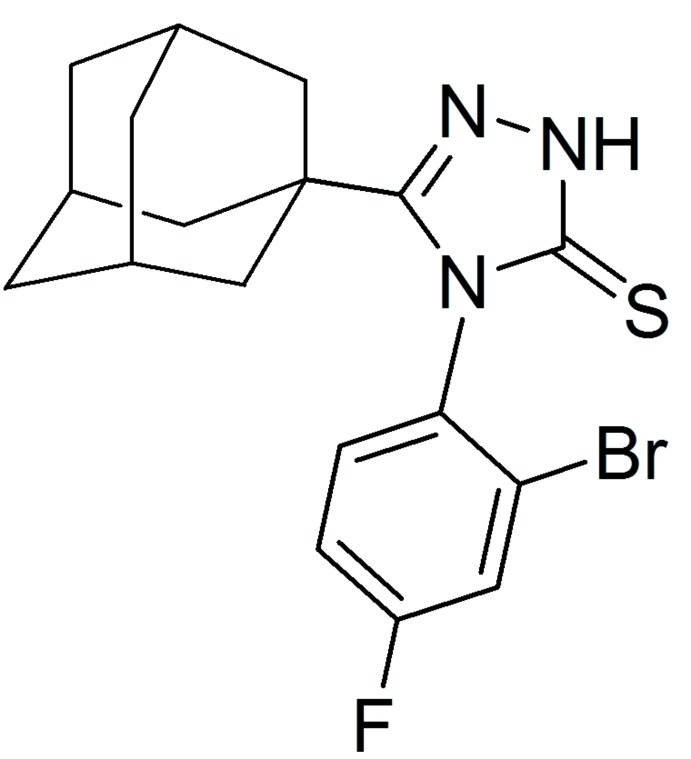


## Structural commentary   

**Figure 1:**
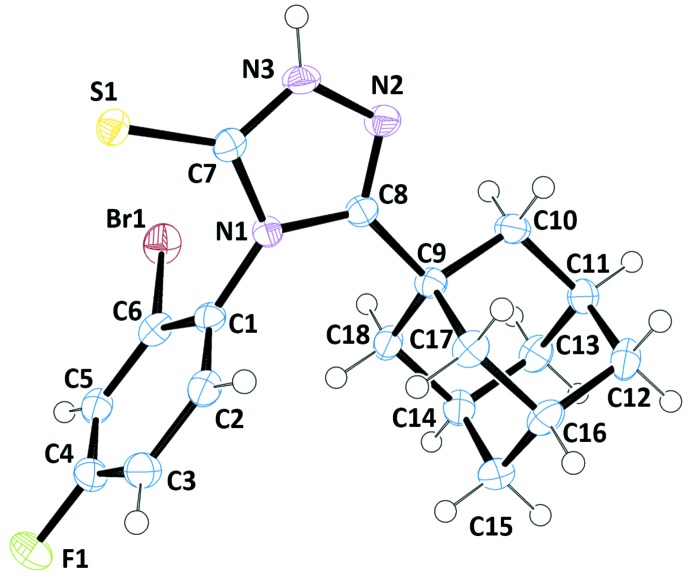
The mol­ecular structure of the title compound with atom labels, showing displacement ellipsoids at the 50% probability level.

In the title mol­ecule (Fig. 1 ▸), the 1,2,4-triazole ring (N1–N3/C7/C8) is nearly planar with a maximum deviation of −0.009 (3) Å for atom C7 and 0.009 (4) Å for atom N1. The phenyl ring (C1–C6) is almost perpendicular to the 1,2,4-triazole ring, forming a dihedral angle of 89.5 (2)°. The triazole ring is substituted in positions 3 and 5 with an adamantane group and a sulfur atom which deviate from the mean plane of the ring of −0.149 (4) and −0.067 (1) Å, respectively. The phenyl group is substituted at positions 2 and 4 by a bromine and a fluorine atom, which deviate by 0.001 (4) and 0.014 (2) Å, respectively, from the ring plane. The bond distances are in normal ranges for this type of compound [C4—F1 = 1.355 (4), C6—Br1 = 1.898 (3) and C7—S1 = 1.688 (3) Å]. The double-bond character of the C8=N2 bond is evidenced by its length of 1.288 (4) Å, while the other distances in the triazole ring are indicative of electronic delocalization [N2—N3, C7—N3 and C7—N1 = 1.377 (4), 1.327 (4) and 1.379 (4) Å, respectively].

## Supra­molecular features   

**Figure 2:**
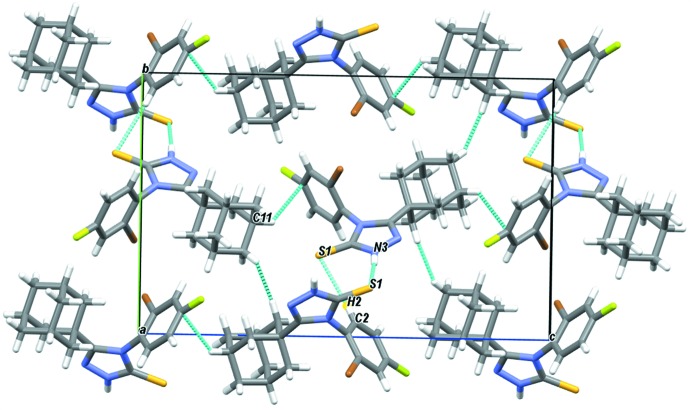
A view of the packing of **I** along the *a*-axis direction. The N—H⋯S hydrogen bonds and C—H⋯S and C—H⋯π inter­actions are shown as dashed lines.

In the crystal (Fig. 2 ▸), the mol­ecules are linked by weak inter­action of the type C–H⋯π(phen­yl), forming supra­molecular chains extending along the *c-*axis direction, involving the C11–H11 group of the adamantane moiety and the C1–C6 aromatic ring. The crystal packing is further consolidated by inter­molecular N3—H3*N*⋯S1(*x* − 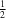, −*y* + 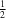, −*z* + 1) hydrogen bonds, in which the triazole ring behaves both as donor and acceptor, and by weak C2—H2⋯S1(*x* + 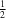, −*y* + 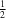, −*z* + 1) inter­actions (Table 1 ▸), yielding double chains propagating along the *a*-axis direction.

## Hirshfeld surface analysis   

**Figure 3:**
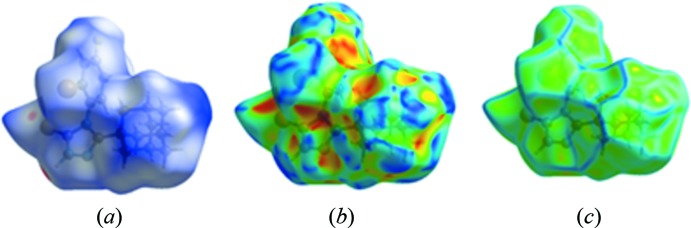
Hirshfeld surfaces of compound **I**, plotted over (*a*) *d*_norm_, (*b*) shape-index and (*c*) curvedness.

In order to investigate the inter­molecular inter­actions in the structure of **I** in a visual manner, a Hirshfeld surface analysis was performed using the program *Crystal Explorer 17.5* (Spackman *et al.* 2002 ▸; Turner *et al.*, 2017 ▸). Fig. 3 ▸ shows the HS surfaces mapped over *d*_norm_, shape-index and curvedness (Fig. 3 ▸). In the HS plotted over *d*_norm_, white areas on the surface indicate contacts with distances equal to the sum of van der Waals radii, and the red and blue colours indicate distances shorter (in close contact) or longer (distant contact) than the van der Waals radii, respectively. Two red spots are present in close proximity to the S and N—H atoms involved in hydrogen bonding. As expected, the absence of red and blue triangles on the shape-index surface and the small, flat segments delineated by the blue line in the surface mapped over curvedness indicate the absence of π–π stacking inter­actions in the crystal structure, while the red regions over the shape-index surface are due to the presence of C—H⋯π inter­actions.

**Figure 4:**
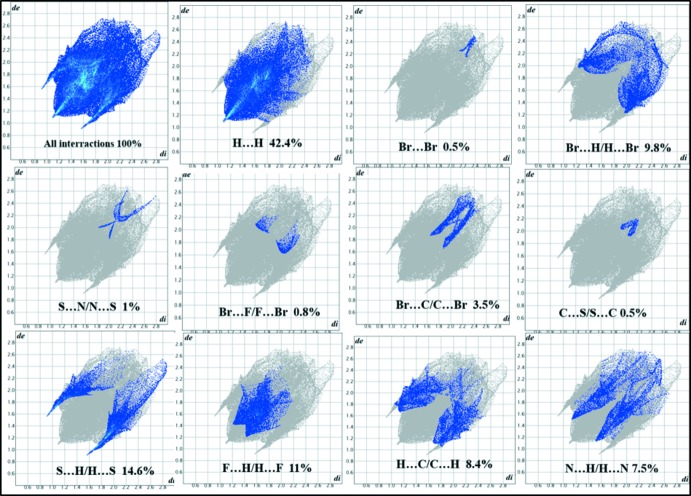
Fingerprint plots of the major inter­actions in compound **I**.

The two-dimensional fingerprint maps for **I** provide some qu­anti­tative information about the individual contributions of the inter­molecular inter­actions in the asymmetric unit (Figs. 4 ▸ and 5 ▸); the distinct spikes appearing in these plots help estimate the different inter­action motifs in the crystal packing. As can be seen from Fig. 4 ▸, no C⋯C inter­actions are present, which confirms the absence of π–π stacking in **I**. Globally, the highest contribution to the total Hirshfeld surface comes from the H⋯H (42.4%) and S⋯H/H⋯S (14.6%) inter­molecular contacts. This indicates that van der Waals forces have an important influence on the consolidation of the crystal structure. The other contacts contribute less to the Hirshfeld surfaces: F⋯H/H⋯F (11%), Br⋯H/H⋯Br (9.8%), H⋯C/C⋯H (8.4%), N⋯H/H⋯N (7.5%), Br⋯C/C⋯Br (3.5%), S⋯N/N⋯S (1%), Br⋯Br (0.5%) and C⋯S/S⋯C (0.5%).

## Database survey   

A search of the Cambridge Structural Database (Version 2.0.1, last update, February 2019; Groom *et al.*, 2016 ▸) for adamantyl triazole-5(4*H*)-thione derivatives gave six hits containing a substituted triazole ring, namely: 3-(adamantan-1-yl)-4-benzyl-1*H*-1,2,4-triazole-5(4*H*)-thione (XOFLEL; Al-Omary *et al.*, 2014 ▸), with a benzyl substituent at position 4 of the 1,2,4-triazole ring; 3-(adamantan-1-yl)-4-(4-fluoro­phen­yl)-1*H*-1,2,4-triazole-5(4*H*)-thione (JAWZUF; Al-Shehri *et al.*, 2017 ▸), in which an F atom is the only substituent on the phenyl ring in the *para* position; 3-(adamantan-1-yl)-4-(prop-2-en-1-yl)-1*H*-1,2,4-triazole-5(4*H*)-thione (LANXAB; Almutairi *et al.*, 2012 ▸), which exhibits a propenyl group, instead of a phenyl one, at position 4 of the triazole ring; 3-(adamantan-1-yl)-4-ethyl-1*H*-1,2,4-triazole-5(4*H*)-thione (ZAPJUX; El-Emam *et al.*, 2012 ▸), which has an ethyl group instead of a phenyl ring at position 4 of the triazole ring; 3-(adamantan-1-yl)-4-(4-chloro­phen­yl)-1*H*-1,2,4-triazole-5(4*H*)-thione (WOTQUT; Al-Wabli *et al.*, 2015 ▸), with a Cl atom in the *para* position of the phenyl ring attached to the triazole moiety; 5-(adamantan-1-yl)-4-phenyl-2,4-di­hydro-1,2,4-triazole-3-thione (WUM­PUP; Nieger *et al.*, 2002 ▸), comprising a phenyl ring, without any substituents, at position 4 of the triazole ring. All of the substituents at position 4 of the planar triazole ring in these compounds are almost perpendicular to that ring, similar to the orientation of the phenyl substituent of the title compound. In the structures of all these compounds, the N—H⋯S inter­actions play an important role in consolidating the crystal packing, along with C–H⋯π inter­actions, when phenyl groups are present as substituents.

## Synthesis and crystallization   

**Figure 6:**
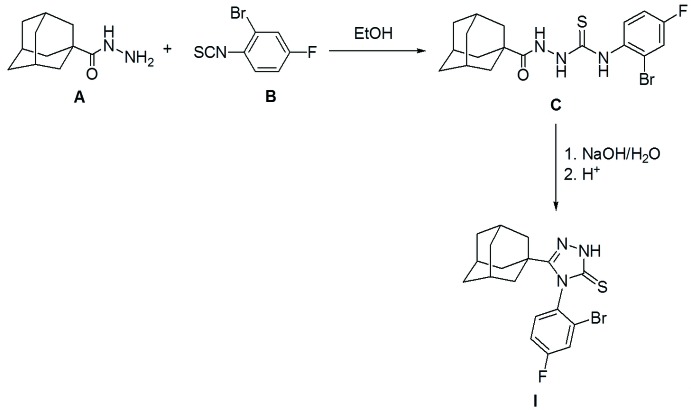
The reaction scheme yielding compound **I**.

All chemicals and solvents were used as purchased without further purification. The melting point was determined using an electrothermal digital melting-point apparatus and uncorrected. The NMR spectra were recorded at room temperature in DMSO-*d*_6_ solution on a Bruker Ascend 700 NMR spectrometer. The title compound **I** was synthesized starting with adamantane-1-carbohydrazide **A** (El-Emam & Ibrahim, 1991 ▸) *via* the reaction with 2-bromo-4-fluoro­phenyl iso­thio­cyanate **B** to yield the corresponding 4-(1-adamantylcarbon­yl)-1-(2-bromo-4-fluoro­phen­yl)-2-thio­semicarbazide **C**, which was then cyclized to the title compound **I** by heating in aqueous sodium hydroxide as outlined in Fig. 6 ▸.

2-Bromo-4-fluoro­phenyl iso­thio­cyanate (2.32 g, 0.01 mol) was added to a solution of adamantane-1-carbohydrazide (1.94 g, 0.01 mol), in ethanol (10 mL), and the mixture was heated under reflux with stirring for one h. Ethanol was then distilled off *in vacuo* and an aqueous sodium hydroxide solution (10%, 15 mL) was added to the residue and the mixture was heated under reflux for 4 h, then filtered hot. On cooling, the mixture was acidified with hydro­chloric acid (*p*H 1–2) and the precipitated crude product was filtered, washed with water, dried and crystallized from an aqueous medium to yield 3.06 g (75%) of the title compound (C_18_H_19_BrFN_3_S) as fine colourless crystals (m.p. 577–579 K). Single crystals suitable for X-ray diffraction were obtained by slow evaporation of a solution of the title compound in EtOH/CHCl_3_ (1:2, *v*/*v)* at room temperature. ^1^H NMR (DMSO-*d*_6_, 700.17 MHz): δ 1.47–1.71 (*m*, 9H, adamantane-H), 1.87–1.89 (*s*, 6H, adamantane-H), 7.47–7.52 (*m*, 1H, Ar-H), 7.69–7.71 (*m*, 1H, Ar-H), 7.87–7.90 (*m*, 1H, Ar-H), 13.86 (*s*, 1H, NH). ^13^C NMR (DMSO-*d*_6_, 176.08 MHz): δ 27.92, 36.08, 36.57, 38.49 (adamantane-C), 115.0, 121.15, 131.65, 134.16, 135.21, 161.86 (Ar-C), 159.46 (triazole C=N), 169.41 (triazole C=S).

## Refinement   

Crystal data, data collection and structure refinement details are summarized in Table 2 ▸. Carbon and nitro­gen-bound H atoms were placed in calculated positions (C—H 0.95 to 0.98 Å; N—H 0.86 Å) and were included in the refinement in the riding-model approximation, with *U*_iso_(H) set to 1.2 to 1.5*U*_eq_(C,N). The structure was refined as a racemic twin [BASF: 0.50 (2)]. Four reflections (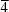 12 10, 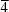 12 5, 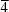 12 6 and 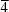 12 9) were omitted from the last cycle of refinement owing to poor agreement.

## Supplementary Material

Crystal structure: contains datablock(s) global, I. DOI: 10.1107/S2056989020000092/xi2020sup1.cif

Structure factors: contains datablock(s) I. DOI: 10.1107/S2056989020000092/xi2020Isup2.hkl

CCDC reference: 1975689

Additional supporting information:  crystallographic information; 3D view; checkCIF report

## Figures and Tables

**Figure 5:**
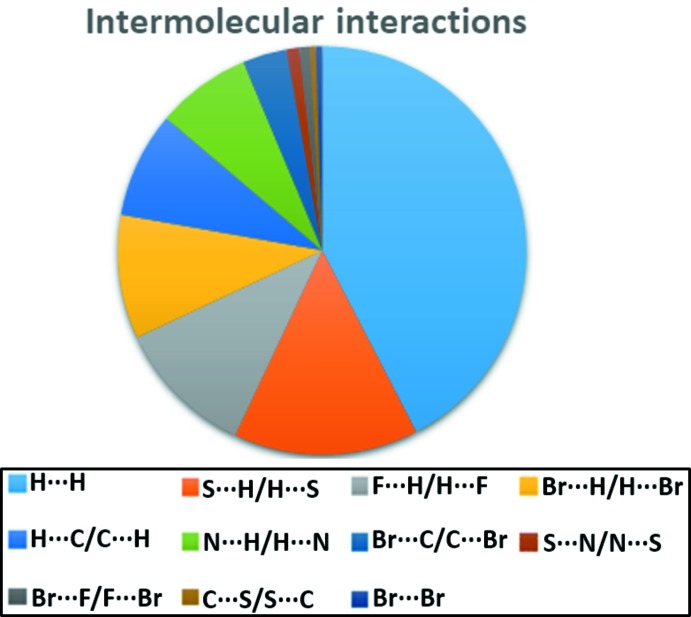
Relative contribution of the various inter­molecular inter­actions in compound **I**.

**Table 1:** Hydrogen-bond geometry (Å, °) *Cg*2 is the centroid of the C1–C2 ring.

*D*—H⋯*A*	*D*—H	H⋯*A*	*D*⋯*A*	*D*—H⋯*A*
N3—H3*N*⋯S1^i^	0.86	2.62	3.461 (3)	166
C2—H2⋯S1^ii^	0.93	2.8	3.683 (3)	160
C11—H11⋯C*g*2^iii^	0.98	2.86	3.757 (4)	152

Symmetry codes: (i) 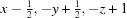; (ii) 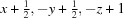; (iii) 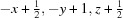.

**Table 2:** Experimental details

Crystal data	
Chemical formula	C_18_H_19_BrFN_3_S
*M*_r_	408.32
Crystal system, space group	Orthorhombic, *P*2_1_2_1_2_1_
Temperature (K)	151
*a*, *b*, *c* (Å)	6.8473 (1), 12.5587 (2), 19.8090 (3)
*V* (Å^3^)	1703.44 (4)
*Z*	4
Radiation type	Cu *K*α
μ (mm^−1^)	4.56
Crystal size (mm)	0.12 × 0.11 × 0.08

Data collection	
Diffractometer	Agilent Excalibur, Ruby, Gemini
Absorption correction	Multi-scan (*CrysAlis PRO*; Agilent 2014 ▸)
*T*_min_, *T*_max_	0.602, 0.694
No. of measured, independent and observed [*I* > 2σ(*I*)] reflections	13191, 3572, 3244
*R*_int_	0.046
(sin θ/λ)_max_ (Å^−1^)	0.630

Refinement	
*R*[*F* ^2^ > 2σ(*F* ^2^)], *wR*(*F* ^2^), *S*	0.035, 0.081, 1.05
No. of reflections	3498
No. of parameters	218
H-atom treatment	H-atom parameters constrained
Δρ_max_, Δρ_min_ (e Å^−3^)	0.62, −0.36
Absolute structure	Flack (1983 ▸)
Absolute structure parameter	0.50 (2)

Computer programs: *SIR2011* (Burla *et al.*, 2012 ▸), *SHELXL* (Sheldrick, 2015 ▸), *ORTEP-3 for Windows* and *WinGX* (Farrugia, 2012 ▸), *Mercury* (Macrae *et al.*, 2008 ▸), *SCHAKAL* (Keller, 1989 ▸), *DIAMOND* (Brandenburg, 2006 ▸), *PARST* (Nardelli, 1995 ▸), *publCIF* (Westrip, 2010 ▸), *enCIFer* (Allen *et al.*, 2004 ▸) and *PLATON* (Spek, 2009 ▸).

## supplementary crystallographic information

### Crystal data

**Table:**

C_18_H_19_BrFN_3_S	*F*(000) = 832
*M**_r_* = 408.32	*D*_x_ = 1.592 Mg m^−^^3^
Orthorhombic, *P*2_1_2_1_2_1_	Cu *K*α radiation, λ = 1.54184 Å
Hall symbol: P 2ac 2ab	Cell parameters from 5537 reflections
*a* = 6.8473 (1) Å	θ = 4.2–74.6°
*b* = 12.5587 (2) Å	µ = 4.56 mm^−^^1^
*c* = 19.8090 (3) Å	*T* = 151 K
*V* = 1703.44 (4) Å^3^	Prism, colourless
*Z* = 4	0.12 × 0.11 × 0.08 mm

### Data collection

**Table:**

Agilent Excalibur, Ruby, Gemini diffractometer	3572 independent reflections
Graphite monochromator	3244 reflections with *I* > 2σ(*I*)
Detector resolution: 10.2673 pixels mm^-1^	*R*_int_ = 0.046
ω scans	θ_max_ = 76.3°, θ_min_ = 4.2°
Absorption correction: multi-scan (CrysAlisPro; Agilent 2014)	*h* = −8→8
*T*_min_ = 0.602, *T*_max_ = 0.694	*k* = −15→14
13191 measured reflections	*l* = −23→24

### Refinement

**Table:**

Refinement on *F*^2^	Secondary atom site location: difference Fourier map
Least-squares matrix: full	Hydrogen site location: inferred from neighbouring sites
*R*[*F*^2^ > 2σ(*F*^2^)] = 0.035	H-atom parameters constrained
*wR*(*F*^2^) = 0.081	*w* = 1/[σ^2^(*F*_o_^2^) + (0.0337*P*)^2^ + 1.0546*P*] where *P* = (*F*_o_^2^ + 2*F*_c_^2^)/3
*S* = 1.05	(Δ/σ)_max_ = 0.001
3498 reflections	Δρ_max_ = 0.62 e Å^−^^3^
218 parameters	Δρ_min_ = −0.36 e Å^−^^3^
0 restraints	Absolute structure: Flack (1983)
0 constraints	Absolute structure parameter: 0.50 (2)
Primary atom site location: structure-invariant direct methods	

### Special details

**Table:**

Geometry. All esds (except the esd in the dihedral angle between two l.s. planes) are estimated using the full covariance matrix. The cell esds are taken into account individually in the estimation of esds in distances, angles and torsion angles; correlations between esds in cell parameters are only used when they are defined by crystal symmetry. An approximate (isotropic) treatment of cell esds is used for estimating esds involving l.s. planes.
Refinement. Refinement of F^2^ against ALL reflections. The weighted R-factor wR and goodness of fit S are based on F^2^, conventional R-factors R are based on F, with F set to zero for negative F^2^. The threshold expression of F^2^ > 2sigma(F^2^) is used only for calculating R-factors(gt) etc. and is not relevant to the choice of reflections for refinement. R-factors based on F^2^ are statistically about twice as large as those based on F, and R- factors based on ALL data will be even larger.

### Fractional atomic coordinates and isotropic or equivalent isotropic displacement parameters (Å^2^)

**Table:**

	*x*	*y*	*z*	*U*_iso_*/*U*_eq_	
Br1	−0.03402 (6)	0.64125 (3)	0.484532 (19)	0.03206 (11)	
S1	−0.01416 (12)	0.31086 (6)	0.43635 (4)	0.02208 (17)	
F1	0.6045 (3)	0.6344 (2)	0.34672 (10)	0.0370 (5)	
N1	0.1505 (4)	0.4307 (2)	0.53736 (13)	0.0178 (6)	
N3	−0.0802 (4)	0.3300 (2)	0.57154 (14)	0.0247 (7)	
H3N	−0.1756	0.2854	0.572	0.03*	
N2	−0.0120 (4)	0.3805 (2)	0.62852 (12)	0.0239 (6)	
C4	0.4956 (5)	0.5857 (3)	0.39470 (15)	0.0251 (8)	
C1	0.2724 (5)	0.4870 (3)	0.49068 (16)	0.0202 (6)	
C5	0.3203 (5)	0.6311 (3)	0.41180 (15)	0.0240 (7)	
H5	0.2778	0.6939	0.3917	0.029*	
C7	0.0153 (4)	0.3567 (2)	0.51580 (14)	0.0195 (6)	
C8	0.1279 (5)	0.4408 (3)	0.60780 (16)	0.0202 (7)	
C11	0.2990 (5)	0.5637 (3)	0.77521 (16)	0.0256 (8)	
H11	0.2373	0.5644	0.8198	0.031*	
C2	0.4508 (5)	0.4436 (3)	0.47200 (15)	0.0238 (7)	
H2	0.4943	0.3811	0.4922	0.029*	
C17	0.4578 (5)	0.4462 (3)	0.65943 (16)	0.0242 (7)	
H17A	0.4376	0.3733	0.6741	0.029*	
H17B	0.5197	0.4446	0.6154	0.029*	
C3	0.5643 (5)	0.4932 (3)	0.42340 (16)	0.0273 (8)	
H3A	0.6839	0.4647	0.4105	0.033*	
C6	0.2089 (5)	0.5793 (3)	0.46035 (16)	0.0212 (7)	
C14	0.4279 (5)	0.6766 (3)	0.68164 (16)	0.0260 (8)	
H14	0.4493	0.7499	0.6663	0.031*	
C9	0.2594 (5)	0.5037 (3)	0.65435 (16)	0.0200 (7)	
C12	0.4946 (6)	0.5054 (3)	0.77950 (15)	0.0280 (8)	
H12A	0.5792	0.5411	0.8116	0.034*	
H12B	0.4739	0.433	0.795	0.034*	
C16	0.5909 (5)	0.5041 (3)	0.70996 (17)	0.0269 (8)	
H16	0.7167	0.4671	0.7127	0.032*	
C10	0.1647 (5)	0.5061 (3)	0.72489 (16)	0.0254 (8)	
H10A	0.04	0.5425	0.7225	0.031*	
H10B	0.1414	0.4338	0.7402	0.031*	
C13	0.3326 (6)	0.6782 (3)	0.75170 (17)	0.0271 (8)	
H13A	0.4168	0.715	0.7834	0.033*	
H13B	0.2091	0.7159	0.7496	0.033*	
C18	0.2933 (5)	0.6190 (3)	0.63142 (16)	0.0245 (8)	
H18A	0.1692	0.656	0.6287	0.029*	
H18B	0.3523	0.6193	0.5869	0.029*	
C15	0.6237 (5)	0.6188 (3)	0.68563 (18)	0.0298 (9)	
H15A	0.7097	0.6558	0.7167	0.036*	
H15B	0.6852	0.6182	0.6415	0.036*	

### Atomic displacement parameters (Å^2^)

**Table:**

	*U*^11^	*U*^22^	*U*^33^	*U*^12^	*U*^13^	*U*^23^
Br1	0.02922 (18)	0.0329 (2)	0.03404 (17)	0.01027 (18)	−0.00194 (16)	0.00029 (16)
S1	0.0243 (4)	0.0216 (4)	0.0204 (3)	−0.0005 (4)	−0.0022 (3)	−0.0017 (3)
F1	0.0388 (12)	0.0395 (14)	0.0329 (11)	−0.0117 (12)	0.0067 (9)	0.0123 (11)
N1	0.0206 (14)	0.0149 (14)	0.0178 (12)	−0.0008 (12)	−0.0001 (10)	−0.0009 (10)
N3	0.0226 (15)	0.0257 (16)	0.0260 (14)	−0.0095 (12)	0.0020 (11)	−0.0048 (11)
N2	0.0234 (16)	0.0259 (16)	0.0225 (12)	−0.0050 (13)	0.0030 (11)	−0.0077 (10)
C4	0.030 (2)	0.0235 (17)	0.0221 (14)	−0.0089 (16)	0.0000 (14)	0.0023 (12)
C1	0.0212 (15)	0.0185 (16)	0.0210 (14)	−0.0039 (13)	−0.0024 (13)	0.0008 (13)
C5	0.0306 (18)	0.0186 (18)	0.0227 (15)	−0.0049 (17)	−0.0059 (13)	0.0024 (14)
C7	0.0179 (14)	0.0171 (14)	0.0235 (13)	0.0035 (15)	−0.0034 (13)	0.0020 (13)
C8	0.0205 (16)	0.0184 (18)	0.0218 (15)	−0.0009 (14)	−0.0008 (12)	−0.0014 (13)
C11	0.0280 (18)	0.030 (2)	0.0188 (15)	−0.0016 (16)	0.0002 (14)	−0.0028 (14)
C2	0.0253 (16)	0.0234 (17)	0.0226 (15)	−0.0003 (16)	−0.0038 (14)	0.0003 (12)
C17	0.0240 (16)	0.0225 (17)	0.0263 (15)	0.0027 (16)	−0.0016 (15)	−0.0050 (13)
C3	0.0251 (19)	0.031 (2)	0.0262 (16)	−0.0038 (16)	0.0019 (14)	−0.0032 (14)
C6	0.0200 (16)	0.0182 (17)	0.0253 (15)	0.0037 (14)	−0.0033 (13)	−0.0059 (13)
C14	0.035 (2)	0.0182 (17)	0.0249 (16)	−0.0062 (15)	−0.0046 (14)	0.0012 (13)
C9	0.0212 (17)	0.0181 (17)	0.0207 (15)	−0.0021 (14)	0.0007 (12)	−0.0029 (13)
C12	0.037 (2)	0.0249 (18)	0.0226 (14)	−0.0001 (17)	−0.0091 (15)	0.0019 (12)
C16	0.0194 (18)	0.031 (2)	0.0301 (17)	0.0041 (15)	−0.0045 (13)	−0.0030 (15)
C10	0.0231 (18)	0.029 (2)	0.0243 (16)	−0.0059 (16)	0.0038 (13)	−0.0030 (15)
C13	0.0315 (19)	0.023 (2)	0.0266 (17)	0.0010 (16)	−0.0042 (14)	−0.0079 (14)
C18	0.0268 (18)	0.025 (2)	0.0220 (15)	−0.0018 (15)	−0.0063 (13)	0.0010 (13)
C15	0.0246 (18)	0.035 (3)	0.0296 (17)	−0.0081 (17)	−0.0017 (14)	0.0007 (16)

### Geometric parameters (Å, º)

**Table:**

Br1—C6	1.898 (3)	C17—C9	1.542 (5)
S1—C7	1.688 (3)	C17—H17A	0.97
F1—C4	1.355 (4)	C17—H17B	0.97
N1—C7	1.379 (4)	C3—H3A	0.93
N1—C8	1.410 (4)	C14—C15	1.526 (5)
N1—C1	1.433 (4)	C14—C13	1.534 (5)
N3—C7	1.327 (4)	C14—C18	1.537 (5)
N3—N2	1.377 (4)	C14—H14	0.98
N3—H3N	0.86	C9—C18	1.535 (5)
N2—C8	1.288 (4)	C9—C10	1.541 (4)
C4—C5	1.371 (5)	C12—C16	1.527 (5)
C4—C3	1.376 (5)	C12—H12A	0.97
C1—C6	1.377 (5)	C12—H12B	0.97
C1—C2	1.387 (5)	C16—C15	1.536 (5)
C5—C6	1.389 (5)	C16—H16	0.98
C5—H5	0.93	C10—H10A	0.97
C8—C9	1.512 (5)	C10—H10B	0.97
C11—C12	1.529 (5)	C13—H13A	0.97
C11—C13	1.529 (5)	C13—H13B	0.97
C11—C10	1.537 (5)	C18—H18A	0.97
C11—H11	0.98	C18—H18B	0.97
C2—C3	1.385 (5)	C15—H15A	0.97
C2—H2	0.93	C15—H15B	0.97
C17—C16	1.537 (5)		
			
C7—N1—C8	107.1 (3)	C15—C14—H14	109.3
C7—N1—C1	121.6 (3)	C13—C14—H14	109.3
C8—N1—C1	131.2 (3)	C18—C14—H14	109.3
C7—N3—N2	113.5 (3)	C8—C9—C18	113.7 (3)
C7—N3—H3N	123.3	C8—C9—C10	108.2 (3)
N2—N3—H3N	123.3	C18—C9—C10	108.3 (3)
C8—N2—N3	105.2 (3)	C8—C9—C17	108.6 (3)
F1—C4—C5	117.9 (3)	C18—C9—C17	109.2 (3)
F1—C4—C3	118.9 (3)	C10—C9—C17	108.7 (3)
C5—C4—C3	123.2 (3)	C16—C12—C11	109.5 (3)
C6—C1—C2	119.5 (3)	C16—C12—H12A	109.8
C6—C1—N1	120.9 (3)	C11—C12—H12A	109.8
C2—C1—N1	119.4 (3)	C16—C12—H12B	109.8
C4—C5—C6	117.2 (3)	C11—C12—H12B	109.8
C4—C5—H5	121.4	H12A—C12—H12B	108.2
C6—C5—H5	121.4	C12—C16—C15	109.6 (3)
N3—C7—N1	104.1 (2)	C12—C16—C17	109.7 (3)
N3—C7—S1	129.1 (2)	C15—C16—C17	109.0 (3)
N1—C7—S1	126.8 (2)	C12—C16—H16	109.5
N2—C8—N1	110.1 (3)	C15—C16—H16	109.5
N2—C8—C9	123.8 (3)	C17—C16—H16	109.5
N1—C8—C9	125.8 (3)	C11—C10—C9	110.2 (3)
C12—C11—C13	109.6 (3)	C11—C10—H10A	109.6
C12—C11—C10	109.5 (3)	C9—C10—H10A	109.6
C13—C11—C10	109.6 (3)	C11—C10—H10B	109.6
C12—C11—H11	109.4	C9—C10—H10B	109.6
C13—C11—H11	109.4	H10A—C10—H10B	108.1
C10—C11—H11	109.4	C11—C13—C14	109.1 (3)
C3—C2—C1	120.2 (3)	C11—C13—H13A	109.9
C3—C2—H2	119.9	C14—C13—H13A	109.9
C1—C2—H2	119.9	C11—C13—H13B	109.9
C16—C17—C9	110.1 (3)	C14—C13—H13B	109.9
C16—C17—H17A	109.6	H13A—C13—H13B	108.3
C9—C17—H17A	109.6	C9—C18—C14	110.1 (3)
C16—C17—H17B	109.6	C9—C18—H18A	109.6
C9—C17—H17B	109.6	C14—C18—H18A	109.6
H17A—C17—H17B	108.2	C9—C18—H18B	109.6
C4—C3—C2	118.4 (3)	C14—C18—H18B	109.6
C4—C3—H3A	120.8	H18A—C18—H18B	108.2
C2—C3—H3A	120.8	C14—C15—C16	109.5 (3)
C1—C6—C5	121.5 (3)	C14—C15—H15A	109.8
C1—C6—Br1	120.8 (3)	C16—C15—H15A	109.8
C5—C6—Br1	117.7 (3)	C14—C15—H15B	109.8
C15—C14—C13	109.5 (3)	C16—C15—H15B	109.8
C15—C14—C18	109.7 (3)	H15A—C15—H15B	108.2
C13—C14—C18	109.7 (3)		

### Hydrogen-bond geometry (Å, º)

*Cg*2 is the centroid of the C1–C2 ring.

**Table:**

*D*—H···*A*	*D*—H	H···*A*	*D*···*A*	*D*—H···*A*
N3—H3*N*···S1^i^	0.86	2.62	3.461 (3)	166
C2—H2···S1^ii^	0.93	2.8	3.683 (3)	160
C11—H11···C*g*2^iii^	0.98	2.86	3.757 (4)	152

Symmetry codes: (i) *x*−1/2, −*y*+1/2, −*z*+1; (ii) *x*+1/2, −*y*+1/2, −*z*+1; (iii) −*x*+1/2, −*y*+1, *z*+1/2.
